# A homologous NADC34-like PRRSV-2 inactivated vaccine partially modulates pulmonary viral load and pathological lesions under controlled challenge conditions

**DOI:** 10.3389/fvets.2026.1932482

**Published:** 2026-10-21

**Authors:** Su-Hwa You, Hyun-Ji Lee, Seung-Min Song, Eun-Gyeong Shin, Jae-Myung Kim, Yeun-Kyung Shin, Seok-Jin Kang

**Affiliations:** Viral Diseases Research Division, Animal and Plant Quarantine Agency, Gyeongsangbuk, Gimcheon, Gyeongsangbuk-do, Republic of Korea

**Keywords:** carbomer-based adjuvant, homologous challenge, inactivated vaccine, NADC34-like strain, PRRSV-2, pulmonary pathology

## Abstract

**Background:**

Porcine reproductive and respiratory syndrome virus (PRRSV) continues to cause substantial economic losses in the global swine industry, largely due to its rapid genetic evolution and capacity for immune evasion. Lineage 1 NADC34-like variants have become increasingly prevalent in East Asia, raising concerns about the effectiveness of existing vaccines against these emerging strains.

**Methods:**

Herein, the effects of an inactivated NADC34-like vaccine strain derived from a contemporary Korean isolate were evaluated under homologous experimental challenge conditions. The vaccine antigen was prepared at a high titer (10^8^ TCID_50_/dose), formulated with different adjuvants, including Carbopol/squalene-based formulations, and administered to 3-week-old pigs, followed by an intranasal challenge with a homologous NADC34-like strain. Clinical parameters, blood and tissue viral loads, humoral and cellular immune responses, and pulmonary pathology were assessed.

**Results:**

Vaccinated pigs, particularly those receiving Carbopol/squalene-based formulations, exhibited reduced body temperature and lung viral loads, and lower microscopic interstitial pneumonia scores compared with nonvaccinated controls. However, no consistent or statistically significant reduction in systemic viral kinetics was observed across the vaccinated groups.

**Discussion:**

These findings indicate that homologous inactivated vaccination does not achieve complete systemic viral suppression but its efficacy is limited to clinical mitigation and the reduction of pulmonary pathological outcomes. The dissociation between systemic viral load and pulmonary pathology highlights the complexity of vaccine evaluation in PRRSV infection. Nevertheless, the alleviation of body temperature, body weight, and lung lesions is of great significance in terms of protective potential against PRRS, a wasting disease, conferred by the inactivated vaccine.

## Introduction

Porcine reproductive and respiratory syndrome virus (PRRSV) remains one of the most economically devastating pathogens affecting the global swine industry, causing severe reproductive failure in sows and respiratory diseases in growing pigs (1). Annual losses in the United States alone are estimated to exceed $664 million, with comparable impacts reported across Europe and Asia.

PRRSV is an enveloped, positive-sense, single-stranded RNA virus with a 15-kb genome containing ≥10 open reading frames (ORFs) and is classified within the family *Arteriviridae* (2). It is divided into two genetically distinct species, *Betaarterivirus suid 1* (PRRSV-1) and *Betaarterivirus suid 2* (PRRSV-2) (3), which share similar clinical manifestations but exhibit ~40% genetic divergence (3, 4). High mutation rates and frequent recombination events drive continuous genetic drift, allowing the virus to effectively evade host immune responses (5). This evolutionary capacity contributes to reduced vaccine effectiveness (4, 6, 7).

PRRSV-2 is phylogenetically classified into nine major lineages based on ORF5 sequences, which encode the major envelope glycoprotein GP5 and serve as a standard molecular marker for PRRSV epidemiological classification (8, 9). Although Lineages 5 and 8 historically predominated in several regions, the global distribution of PRRSV-2 has shifted substantially toward Lineage 1 variants (10). Among these, NADC34-like strains, belonging to the Sublineage 1.5, have emerged as a major concern in East Asia owing to their rapid spread and increased pathogenicity (11). NADC34-like strains are characterized by a continuous 100-amino-acid deletion in the NSP2 region and frequently exhibit recombinant genomic architecture, reflecting ongoing genetic exchange with cocirculating PRRSV lineages (11, 12).

PRRS is wasting disease characterized by interstitial pneumonia with high fever in piglets, as well as abortions and stillbirths in pregnant sows (13). Current vaccination strategies rely primarily on modified live virus (MLV) vaccines. Although MLV vaccines induce robust immune responses, they carry inherent risks, including reversion to virulence and recombination with circulating field strains (6, 7). In contrast, inactivated vaccines offer superior biosafety profiles but have historically exhibited limited efficacy in both homologous and heterologous challenge studies (6, 14). The major MLV vaccines currently utilized in South Korea are Ingelvac PRRS (Boehringer Ingelheim), Prime Pac PRRS (MSD), Fostera PRRS (Zoetis), Unistrain PRRS (Hipra), PoaVac PRRS (Biopoa), Pocillis PRRS (MSD), Immunis PRRS-NA (Woogene B&G) and Prevacent PRRS (Elanco). Regarding inactivated vaccine, only a single product, Progressis (Ceva), has been approved in South Korea; however, its application is largely restricted to boosting immunity within PRRS-positive environments.

Vaccine efficacy is comprehensively assessed through clinical, immunological, virological, and pathological parameters. Clinically, the evaluation focuses on hyperthermia induced by post-infection inflammation and subsequent growth retardation due to reduced feed intake, which represents the most critical economic metric for this wasting disease. Immunologically, while IgG antibody titers are frequently measured, they exhibit a poor correlation with protective immunity. Furthermore, neutralizing antibodies provide limited value for efficacy evaluation due to their delayed induction (typically >1 month post-infection) and characteristically low titers (15, 16). Cellular immunity is additionally assessed by measuring IFN-γ production (17). Virological assessments include the quantification of systemic viremia, nasal viral shedding, and viral loads within the lungs and lymphoid tissues. Pathological evaluations examine macroscopic and histopathological pulmonary lesions. Current MLV and inactivated vaccines do not confer sterilizing immunity or complete viral clearance upon PRRSV infection; instead, they merely provide partial mitigation of these clinical and virological parameters (18–20). Consequently, the primary pragmatic goal of current vaccination strategies is to minimize pulmonary inflammation, thereby preventing weight loss and mitigating economic damage. These limitations are fundamentally driven by the intrinsic properties of PRRSV, notably its rapid mutation kinetics and sophisticated immune evasion strategies.

In South Korea, recent surveillance has revealed an increasing prevalence of NADC34-like variants, raising concerns about the efficacy of currently available commercial vaccines. Despite ongoing field vaccination efforts, the disease continues to spread nationwide. Furthermore, our previous study evaluating two commercial vaccines of PRRSV-2 lineage 5 genotype against NADC34-like variants demonstrated a lack of protective efficacy (data not shown). In this regard, this emergence has highlighted the need for lineage-matched vaccine antigens that more closely reflect the genetic characteristics of circulating field strains (6, 10).

To address the challenges posed by emerging variants, this study evaluated the immunogenicity and disease attenuation of an inactivated vaccine platform based on a high-titer (10^8^ TCID_50_/dose) Korean NADC34-like isolate. Using multiple adjuvant systems, including carbomer-based formulations, the ability of the vaccine to modulate clinical outcomes, viral kinetics across blood and tissue compartments, and both arms of the immune response was examined in a 3-week-old pig model under homologous challenge conditions. The findings indicate that, although complete systemic viral clearance was not achieved, the lineage-matched inactivated vaccine can modulate tissue-specific viral pathogenesis, suggesting a potential strategy to mitigate the impact of NADC34-like variants under field conditions.

## Materials and methods

### Cells and viruses

Two Korean PRRSV-2 isolates, designated prrsv23-8s8n and prrsv23-9s15n, were obtained from domestic swine farms in 2023. Both strains were identified as NADC34-like (Lineage 1, Sublineage 1.5) variants isolated from clinical cases of severe reproductive and respiratory diseases. In this study, prrsv23-8s8n was used as the vaccine antigen, whereas prrsv23-9s15n served as the homologous challenge strain. Both viral strains were propagated from single-plaque purifications.

Viruses were initially propagated in primary porcine alveolar macrophages and subsequently expanded in specialized PRRSV-permissive cell lines, p2sPAM-CD332 and 3D4/31-CD332. These cell lines were rendered susceptible to PRRSV by overexpressing CD163 and CD169 (21). Viral titers were determined as 50% tissue culture infectious dose (TCID_50_/mL) on 3D4/31-CD332 cells using the Reed–Muench method (22).

### Genetic and phylogenetic analyses

Viral RNA was extracted and subjected to next-generation sequencing using the MiSeq Reagent Kit v3 (600-cycle) on the Illumina MiSeq system (Illumina, San Diego, CA, USA). Phylogenetic relationships were inferred from full-length genome sequences and *ORF5* gene sequences (n = 122 total). Multiple sequence alignments were performed using CLC Main Workbench 25.0.3, and phylogenetic trees were constructed using the maximum likelihood method under the general time reversible model, with 1,000 bootstrap replicates to assess node support.

For analysis of NSP2 deletion patterns, NSP2 sequences were extracted from the alignment using the VR2332 reference strain in CLC Main Workbench 25.0.3. Sequences were aligned at the amino acid level and subsequently converted into codon-based nucleotide alignments. Based on the gaps identified in the amino acid alignment, two major deletion patterns were distinguished: NADC30-like and NADC34-like strains, characterized by continuous 111- and 100-amino-acid deletions at NSP positions 321–431 and 329–428, respectively.

Recombination analysis was performed using RDP5 with default parameters, and only recombination events supported by at least five of seven built-in algorithms (RDP, GENECONV, Bootscan, MaxChi, Chimaera, SiScan, and 3Seq) were considered notable. Further validation of recombination breakpoints was conducted using SimPlot version 3.5 with a 200-bp window size and the Kimura (2-parameter) distance model. To verify the identified events, separate phylogenetic trees for recombinant and nonrecombinant regions were constructed using MEGA12. Final determination of recombination events was based on consensus among RDP5, SimPlot, and segment-based phylogenetic analyses.

### Inactivation of PRRSV and antigen preparation

Clarified prrsv23-8s8n viral suspension was concentrated using polyethylene glycol (PEG 8000) precipitation before chemical inactivation. Binary ethylenimine (BEI) was prepared by cyclizing 2-bromoethylamine hydrobromide (Sigma-Aldrich) in 0.3 M NaOH at 37 °C for 1 h. The BEI solution was added to achieve a final concentration of 3 mM, and the mixture was incubated at 37 °C for 24 h with continuous agitation. Residual BEI was neutralized with 1 M sodium thiosulfate (10% v/v) for 2 h at 4 °C. Complete inactivation was confirmed by five consecutive blind passages on 3D4/31-CD332 cells. The absence of cytopathic effects (CPE) and no increase in viral RNA copy numbers relative to background levels were verified by qRT-PCR. Each vaccine dose contained 10^8^ TCID_50_ equivalent before inactivation.

### Vaccine formulations

The inactivated prrsv23-8s8n antigen prepared as described above (10^8^ TCID_50_/dose) was used for vaccine formulation. Each vaccine dose was adjusted to a total volume of 2 mL. Carbopol 974P (0.1%, w/v) (Lubrizol Advanced Materials, Cleveland, OH, USA), designated as carbomer homopolymer type B, was incorporated into a squalene-based oil-in-water (O/W) emulsion and mixed with the vaccine antigen at a 50:50 (v/v) ratio. For the LPS-supplemented group, LPS (20 μg/mL) derived from *Hafnia alvei* strain BA2000346 (Eutilex, Seoul, Republic of Korea) was additionally incorporated into the Carbopol/squalene-based O/W emulsion (23). The IMS1313 group and antigen-only group were prepared using Montanide IMS1313 VG (Seppic, Castres, France) and 1 × PBS, respectively, at a 50:50 (v/v) ratio. All formulations were homogenized for 30 min at room temperature. The formulated vaccines were stored at 4 °C and administered within 24 h of preparation.

### Animals and ethical statement

Thirty 3-week-old PRRSV-seronegative crossbred pigs were used in this study. The sample size was determined based on previous PRRSV challenge studies (24, 25) demonstrating detectable differences in viremia and lung lesion scores. All animal procedures were reviewed and approved by the Institutional Animal Care and Use Committee of the Animal and Plant Quarantine Agency (Approval No. 2020000379). Piglets were randomly assigned to six experimental groups (n = 5 per group).

The groups were defined as follows: Group 1 (G1; Carbopol 974P adjuvant), Group 2 (G2; Carbopol 974P plus *Hafnia alvei* LPS adjuvant), Group 3 (G3; IMS1313 adjuvant), Group 4 (G4; antigen only), Group 5 (G5; challenge control), and Group 6 (G6; negative control). All animals were housed in biosafety level 2 facilities with ad libitum access to feed and water.

### Vaccination and challenge procedures

Vaccinated groups received 2 mL of vaccine intramuscularly into the lateral neck region. A booster vaccination was administered at 2 weeks post-vaccination (wpv) on the contralateral side. At 4 wpv (2 weeks post-booster), pigs in G1–G5 were challenged intranasally with 2 mL of prrsv23-9s15n at 10^6^ TCID_50_/mL (1 mL/nostril). Group 6 received sterile PBS. The challenge inoculum was back-titrated on 3D4/31-CD332 cells to confirm the administered viral dose.

### Clinical observation and sample collection

Clinical signs were monitored daily throughout the study. Rectal temperatures were recorded daily for 3 days following each vaccination and for 10 days post-challenge (dpc). Average daily weight gain (ADWG) was calculated weekly. Blood samples were collected at 0, 1, 2, 3, and 4 wpv and at 7, 14, and 21 dpc. Nasal swabs were collected at 0, 7, 14, and 21 dpc. At 21 dpc, all animals were humanely euthanized by an intramuscular injection of Zoletil^Ⓡ^ (10 mg/kg) and Rompun^Ⓡ^ (10 mg/kg) at a 50:50 (v/v) ratio, followed by euthanasia using an intravenous injection of 10% KCI at 10 weeks of age (21 dpc). Macroscopic lung lesions were scored based on the percentage of consolidation in each pulmonary lobe. Tissue samples were collected and stored at −80 °C for viral quantification. To measure the viral load in tissues, 1 g of tissues and 9 mL of 1 × PBS using a tissue homogenizer. The homogenate was then centrifugated at 4,000 rpm for 10 min at 4 °C. Two hundred microliter of the supernatant was used for RNA extraction. Lung sections were fixed in 10% neutral-buffered formalin for histopathological examination.

### Virological and immunological assays

#### Quantitative real-time PCR

Total RNA was extracted using the Maxwell RSC Viral Total Nucleic Acid Purification kit (Promega, Madison, WI, USA) according to the manufacturer’s instructions. The extracted RNA was used for complementary DNA (cDNA) synthesis. The resulting cDNA was then subjected to multiplex PCR using genotype-specific primers and a one-step kit (GeNet Bio, Daejeon, Korea) to amplify PRRSV-1 and PRRSV-2 under the following conditions: reverse transcription at 50 °C for 20 min (1 cycle), predenaturation at 95 °C for 10 min (1 cycle), followed by denaturation at 95 °C for 10 s and annealing/extension at 60 °C for 30 s (40 cycles). A standard curve was generated from 10-fold serial dilutions of a plasmid containing the target gene. The results, initially measured in copies/mL, were converted to log_10_ TCID_50_/mL.

#### Enzyme-linked immunosorbent assay

PRRSV-specific IgG antibodies were quantified using a commercial enzyme-linked immunosorbent assay kit (IDEXX PRRS X3 Ab Test; IDEXX Laboratories, Westbrook, ME, USA). Samples were considered positive when the sample-to-positive (S/P) ratio was ≥0.4.

#### Virus neutralization test

Neutralization antibody titers were determined using a virus neutralization test performed on 3D4/31-CD332 cells. Virus neutralization titers were expressed as the reciprocal of the highest serum dilution that completely inhibited the CPE. Sera were heat-inactivated at 56 °C for 30 min and serially diluted twofold starting at 1:2. An equal volume of virus (100 TCID_50_) was added, and the mixture was incubated for 1 h at 37 °C before inoculation onto cells.

#### IFN-γ ELISPOT assay

The frequency of PRRSV-specific IFN-γ-secreting cells was quantified using a commercial porcine IFN-γ ELISPOT kit (Mabtech, Nacka Strand, Sweden) and an AID ELISPOT Reader (Advanced Imaging Devices GmbH, Strassberg, Germany). Results were expressed as spot-forming cells per 10^6^ peripheral blood mononuclear cells (PBMCs). PBMCs were stimulated with the homologous challenge strain (prrsv23-9s15n) at a multiplicity of infection of 0.1 for 48 h. Phytohemagglutinin (Sigma-Aldrich) served as a positive control, and unstimulated cells served as a negative control.

### Gross pathology and histopathology

#### Macroscopic lung lesion scoring

At necropsy, macroscopic lung lesions were scored based on the percentage of consolidation in each of the seven pulmonary lobes (left cranial, left middle, left caudal, right cranial, right middle, right caudal, and accessory). The total lesion score (0–100) was calculated as the sum of weighted scores assigned to each lobe, as previously described (26).

#### Histopathology

Tissue samples were fixed in 10% neutral-buffered formalin, embedded in paraffin, and sectioned at 4–5 μm for hematoxylin and eosin (H&E; Sigma-Aldrich) staining. Microscopic lesions, specifically interstitial pneumonia, were evaluated in two lung sections from each animal using a scale of 0 (normal), 1 (minimal), 2 (mild), 3 (moderate), and 4 (severe).

### Statistical analysis

Data were analyzed using two-way ANOVA with repeated measurements, followed by Dunnett’s or Sidak’s multiple-comparison tests, where applicable. Normality was assessed using the Shapiro–Wilk test before parametric analyses, with *p* < 0.05 considered statistically significant.

## Results

### Genomic characterization and phylogenetic analysis of NADC34-like isolates

The complete genome sequences of the two Korean PRRSV-2 isolates, prrsv23-8s8n and prrsv23-9s15n, were determined to characterize their genetic backgrounds and genomic features. Full-length genome sequences were deposited in the NCBI GenBank database under accession numbers PZ169274 and PZ169275, respectively. Phylogenetic analysis with the *ORF5* gene sequences (Figure 1a) and full-length genome sequences (Figure 1b) grouped both isolates within Lineage 1.5 (Sublineage 1.5, NADC34-like).

**Figure 1:**
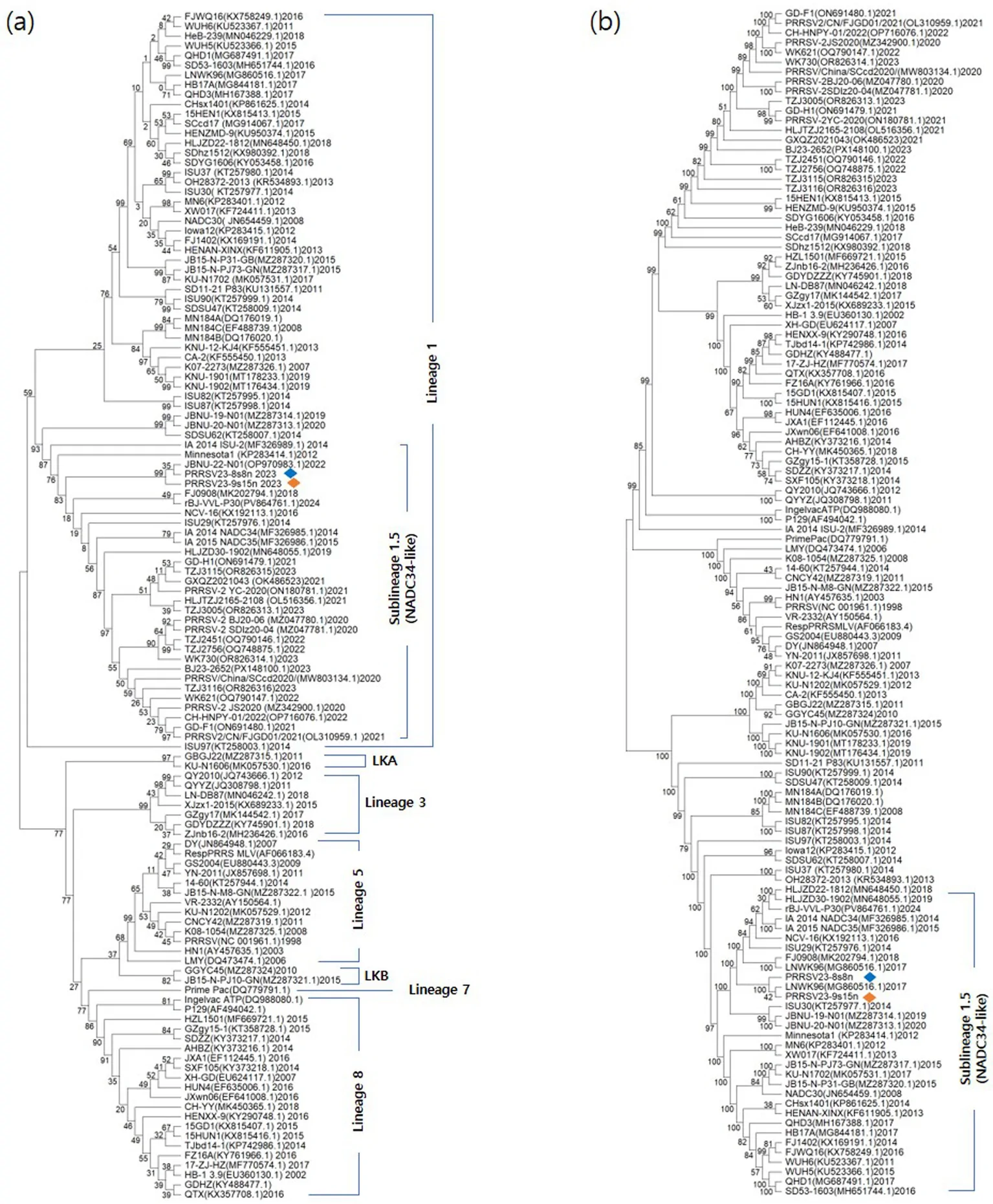
Phylogenetic analysis of Korean PRRSV-2 isolates. The phylogenetic trees of the *ORF5* gene **(a)** and whole-genome sequences **(b)** were constructed using the maximum likelihood method. The two Korean isolates, prrsv23-8s8n (blue diamond) and prrsv23-9s15n (red diamond), clustered within Sublineage 1.5 (NADC34-like), indicating high genetic proximity to the U.S. NADC34 reference strains.

The vaccine candidate and challenge strain shared 96.19 and 95.52% nucleotide identity in *ORF5* with the U.S. NADC34 reference strain (IA/2014/EPICC-01), respectively, and 90.69 and 90.57% whole-genome nucleotide identity, respectively. Between the two isolates, nucleotide identity was 97.01% in *ORF5* and 97.50% across the whole genome (Supplementary Figures 1a,b), with amino acid identities of 98.1 and 95.83%, respectively. Both isolates exhibited the characteristic 100-amino-acid continuous deletion in NSP2 (Figures 2a,b), a molecular signature of the NADC34-like sublineage. Recombination analysis revealed that both isolates possessed mosaic genomic structures incorporating segments derived from VR2332 (Lineage 5) and NADC30-like viruses within an NADC34-like backbone (Figures 2c,d). Recombination events of prrsv23-9s15n were similar with those of prrsv23-8s8n vaccine strain (Supplementary Figure 1c).

**Figure 2:**
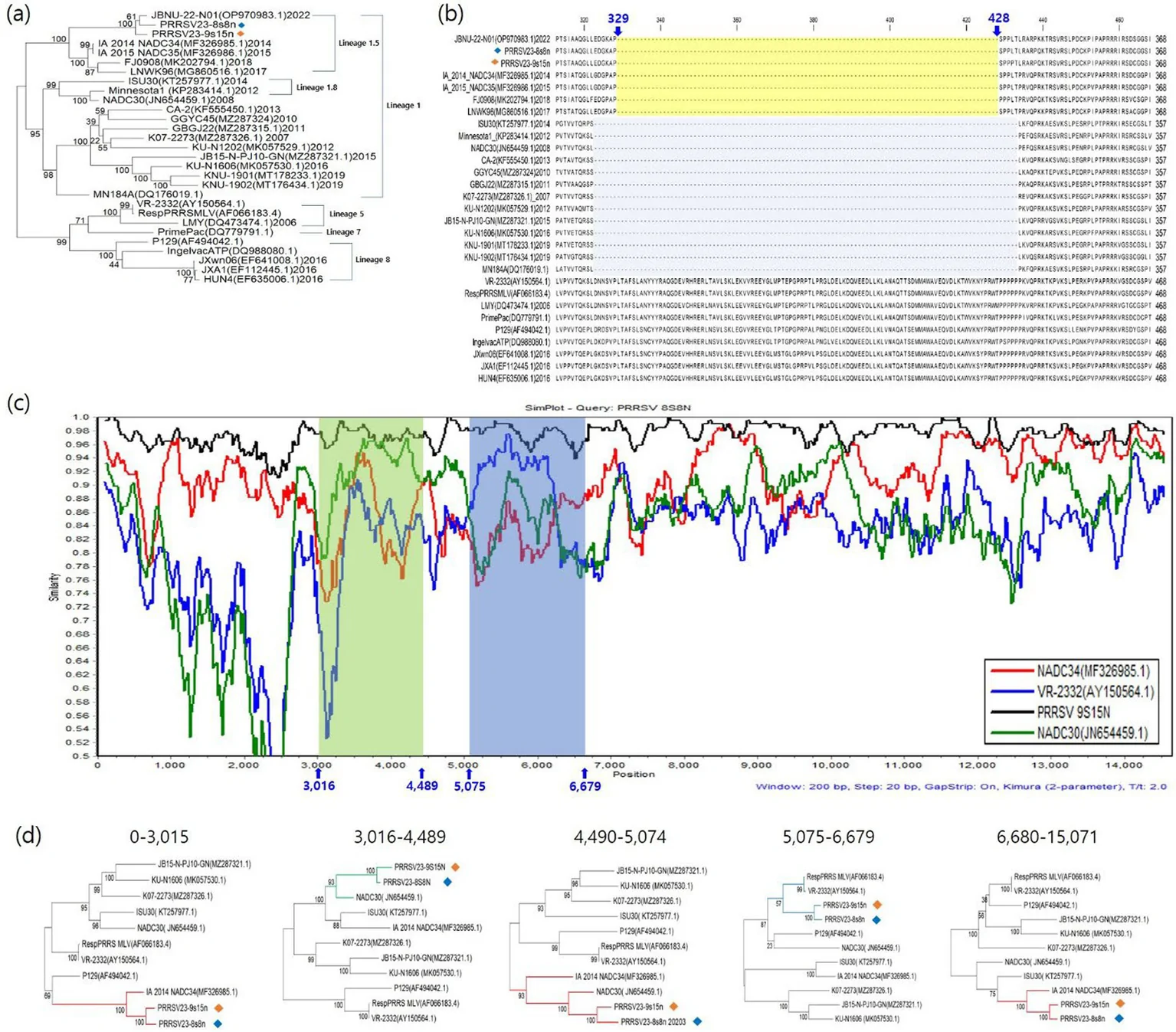
NSP2 characterization and recombination analysis of Korean NADC34-like isolates. **(a)** Phylogenetic analysis of the NSP2 region in the two NADC34-like isolates, prrsv23-8s8n (blue diamond) and prrsv23-9s15n (red diamond). **(b)** Multiple sequence alignment of the NSP2 regions showing that both prrsv23-8s8n and prrsv23-9s15n possess the characteristic 100-amino-acid continuous deletion at a position corresponding to amino acids 329–428 (yellow highlight), a molecular marker of the NADC34-like lineage. **(c)** Recombination events detected in prrsv23-8s8n using RDP5 and SimPlot software. Green (3,016–4,489 nt) and blue (5,075–6,679 nt) regions are recombinant positions of prrsv23-8s8n genome. **(d)** Schematic representation of recombination breakpoints in prrsv23-8s8n, illustrating the incorporation of NADC30-like strain (3,016–4,489 nt) and VR2332 (5,075–6,679 nt) segments into the NADC34-like backbone.

### Clinical signs and growth performance

No vaccine-related local or systemic adverse reactions were observed following primary or booster vaccination, indicating an acceptable safety profile under the experimental conditions. Post-challenge, pigs in G5 exhibited elevated rectal temperatures, with some individuals exceeding 41.0 °C (Figure 3a). Pigs in G1 maintained lower rectal temperatures than those in G5 at several time points post-challenge (*p* < 0.05). Pigs in G2 exhibited a temperature profile similar to that of G5 during the early post-challenge period but showed lower temperatures from 9 dpc onward. No statistically significant differences were observed in G3 or G4 compared with G5. Mean body weight increased steadily across all groups (Figure 3b), with no statistically significant differences among groups. A marked difference in ADWG was observed only between G5 and G6 (Figure 3c). No consistent or statistically significant differences in growth performance were observed between vaccinated groups and the challenge control group.

**Figure 3:**
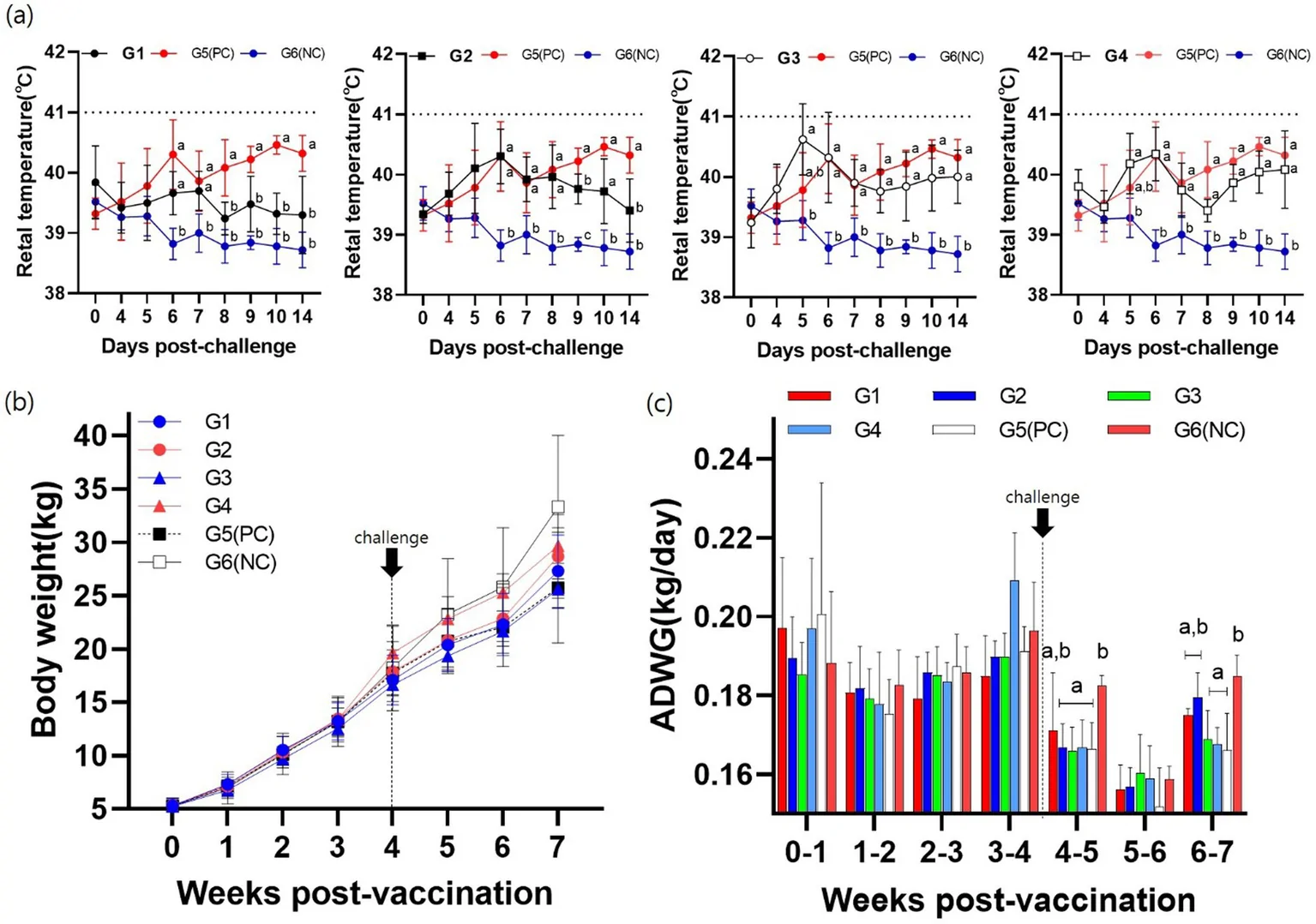
Clinical responses and growth performance following PRRSV-2 challenge. **(a)** Rectal temperature kinetics. Rectal temperatures were monitored daily from 0 to 14 dpc. G1 (Carbopol 974P adjuvant), G2 (Carbopol 974P + LPS adjuvant), G3 (IMS1313 adjuvant), and G4 (antigen only) were compared with G5 (challenge control) and G6 (negative control). **(b)** Changes in body weight. Piglets were weighed weekly throughout the study period to monitor growth trends. **(c)** ADWG. The ADWG was calculated for each group to assess the impact of PRRSV infection on growth performance. Data are presented as mean ± standard deviation. Different letters (a, b, c) indicate statistically significant differences among groups (*p* < 0.05).

### Immunological responses

PRRSV-specific IgG responses were detected in vaccinated groups following immunization (Figure 4a). Seroconversion was observed in G1 at 14 dpv (*p* < 0.05). Post-challenge, all challenged groups exhibited higher S/P ratios than G5 at 4 dpc (*p* < 0.05), with G1 maintaining elevated values up to 7 dpc (*p* < 0.05). Despite the induction of binding antibodies, no neutralizing antibody titers (limit of detection: 1:2) were observed in any group throughout the study. Cell-mediated immune responses, measured by IFN-γ ELISPOT, increased in all challenged groups (Figure 4b). However, no statistically significant differences were observed between vaccinated groups and the challenge control at any time point, indicating that the observed responses were driven primarily by infection rather than vaccination.

**Figure 4:**
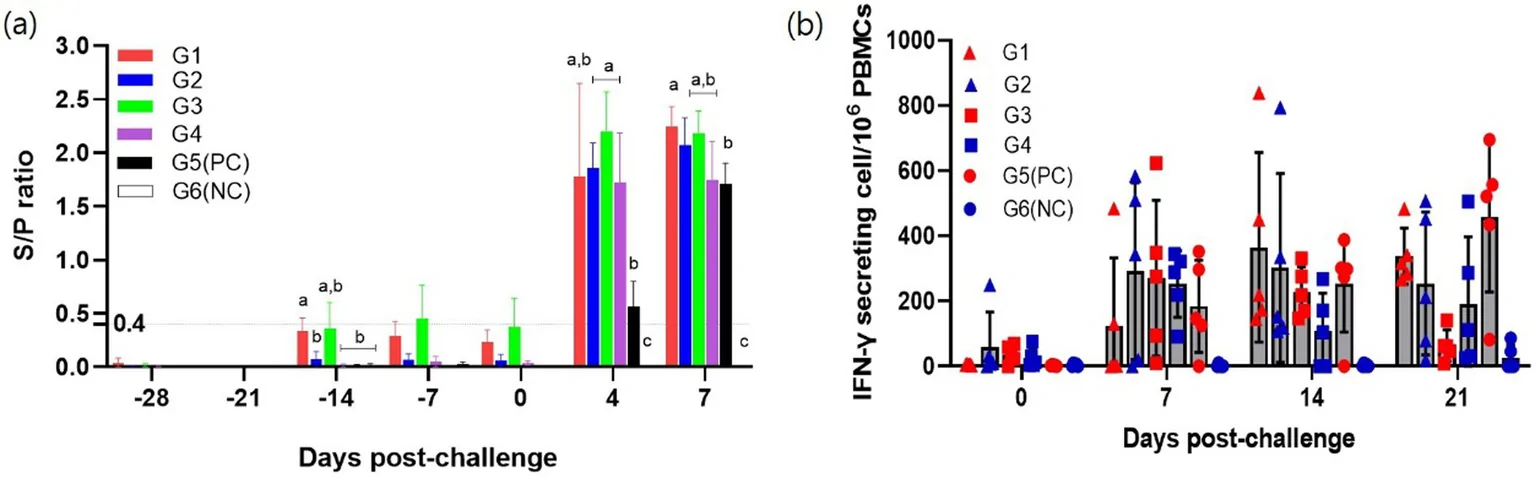
Humoral and cellular immune responses. **(a)** PRRSV-specific IgG antibody responses measured by enzyme-linked immunosorbent assay. Serum S/P ratios were measured from 0 dpv to 7 dpc (35 dpv). Significant seroconversion was observed in G1 and G3 at 14 dpv (*p* < 0.05). Post-challenge, all vaccinated groups (G1–G4) maintained significantly higher S/P ratios than G5 at 4 dpc. Specifically, G1 exhibited the highest S/P ratios, remaining significantly elevated compared with G5 until 7 dpc. Different letters (a, b, c) indicate statistically significant differences among groups (*p* < 0.05). **(b)** PRRSV-specific T-cell responses (IFN-γ ELISPOT). The frequency of IFN-γ-secreting cells was quantified in PBMCs following stimulation with homologous challenge strain prrsv23-9s15n. All vaccinated groups showed an upward trend post-challenge.

### Viremia and viral shedding

Blood viral loads peaked at 7 dpc and declined thereafter in all challenge groups by 21 dpc (Figure 5a and Supplementary Table 1). No consistent or statistically significant reduction of viral load in blood was observed in G1–G4 compared with G5. Notably, blood viral loads in G5 were lower than those reported in previous NADC34-like challenge studies, potentially limiting the ability to detect between-group differences. Viral shedding in nasal swabs was detected in G1–G4 (Figure 5b). Shedding levels in G5 remained relatively low overall, and no statistically significant differences were observed among groups. Although G1 showed no detectable shedding early on, this effect was transient and not sustained during peak shedding periods.

**Figure 5:**
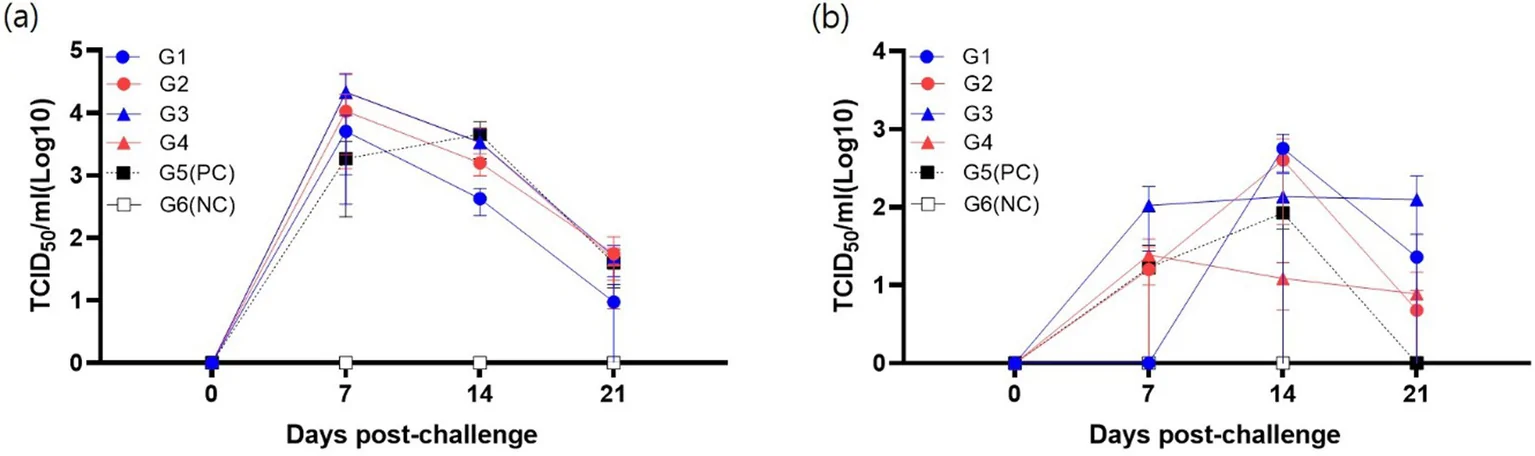
Viremia and nasal shedding kinetics post-challenge. **(a)** Kinetics of systemic viremia post-challenge. Blood viral loads were quantified by RT-qPCR and expressed as log_10_ TCID_50_/mL. Viral loads peaked at 7 dpc and declined by 21 dpc. G1 showed lower titers than the other vaccinated groups; however, G5 also exhibited unexpectedly lower blood viral loads, with no statistically significant differences among groups. **(b)** Evaluation of viral shedding via nasal swabs. Viral loads in nasal swabs were measured post-challenge. Although G1 showed no detectable viral shedding at 7 dpc, no statistically significant differences were observed among any experimental groups throughout the study period.

### Viral loads in tissues

At 21 dpc, viral loads were quantified in lung and lymphoid tissues (Figure 6). A statistically significant reduction in pulmonary viral load was observed only in G1 compared with G5 (*p* < 0.05). G2 showed a numerical reduction that did not reach statistical significance. In contrast, viral loads in the tonsils, mesenteric lymph nodes, and inguinal lymph nodes were comparable across all challenged groups. These findings indicate that detectable effects of vaccination were restricted to pulmonary tissue and were not observed in systemic or other lymphoid compartments.

**Figure 6:**
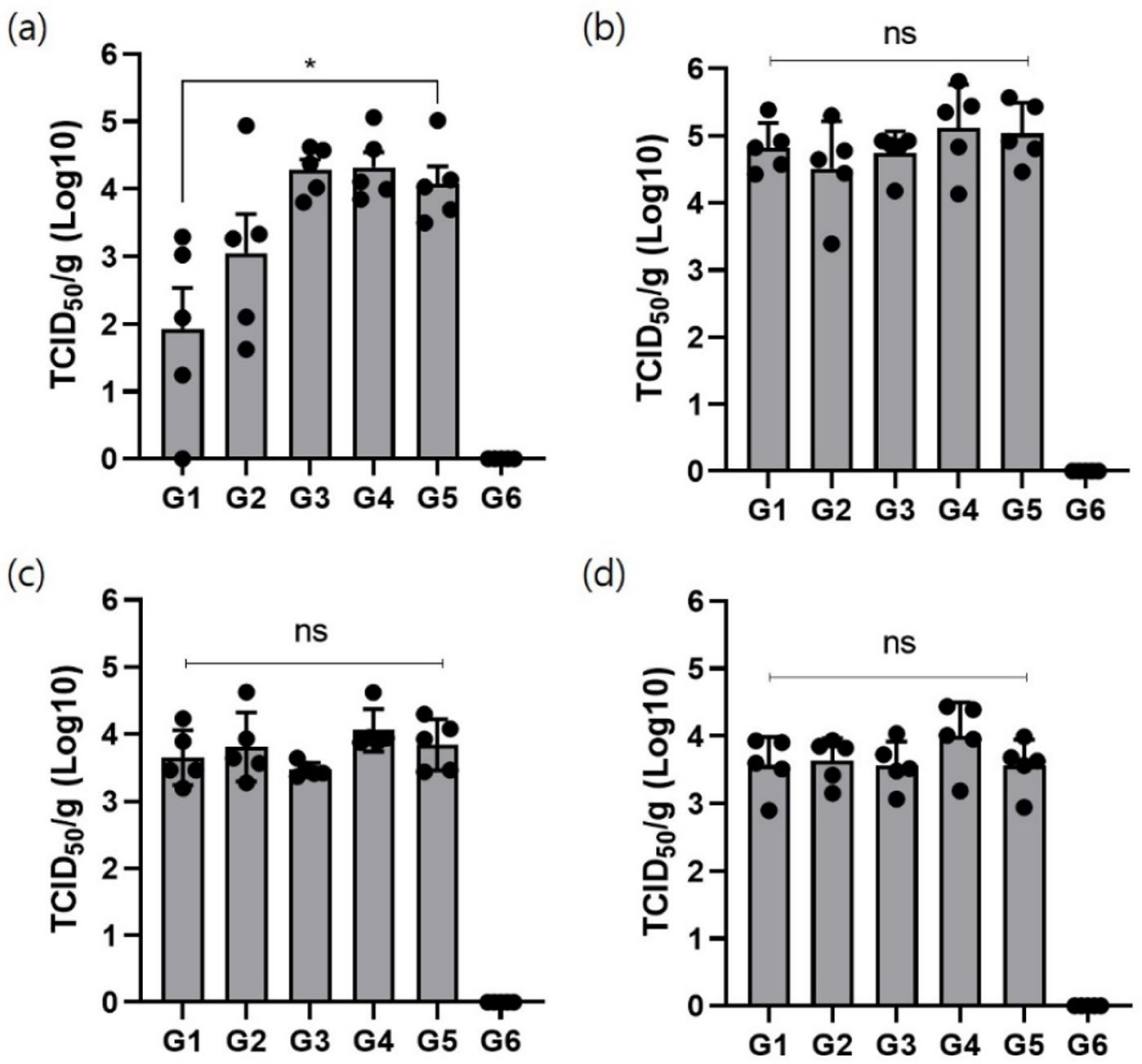
PRRSV-2 viral load in various tissues at 21 dpc. Viral genomic copies were quantified in the lungs **(a)**, tonsils **(b)**, mesenteric lymph nodes **(c)**, and inguinal lymph nodes **(d)**. Tissue viral loads were quantified by RT-qPCR and expressed as log_10_ TCID_50_/g. G1 exhibited a significant reduction (*p < 0.05) in lung viral load compared with G5. No statistically significant differences were observed in tonsils, mesenteric lymph nodes, and inguinal lymph nodes.

### Macroscopic and microscopic lesions

Macroscopic lung lesion scores varied among individual animals (Figures 7a,b). G5 exhibited typical lesions with considerable interindividual variation. G1 showed lower lesion scores compared with G5 (*p* < 0.05). G2 exhibited lower scores; however, the difference was not statistically significant due to within-group variability. Microscopic evaluation revealed interstitial pneumonia across all challenged groups (Figures 7c,d). Significant reductions in microscopic lesion scores were observed in G1 and G2 compared with G5 (*p* < 0.05). These reductions were consistent with the observed decrease in pulmonary viral load; however, the effects were restricted to tissue-level outcomes and were not reflected in systemic virological parameters.

**Figure 7:**
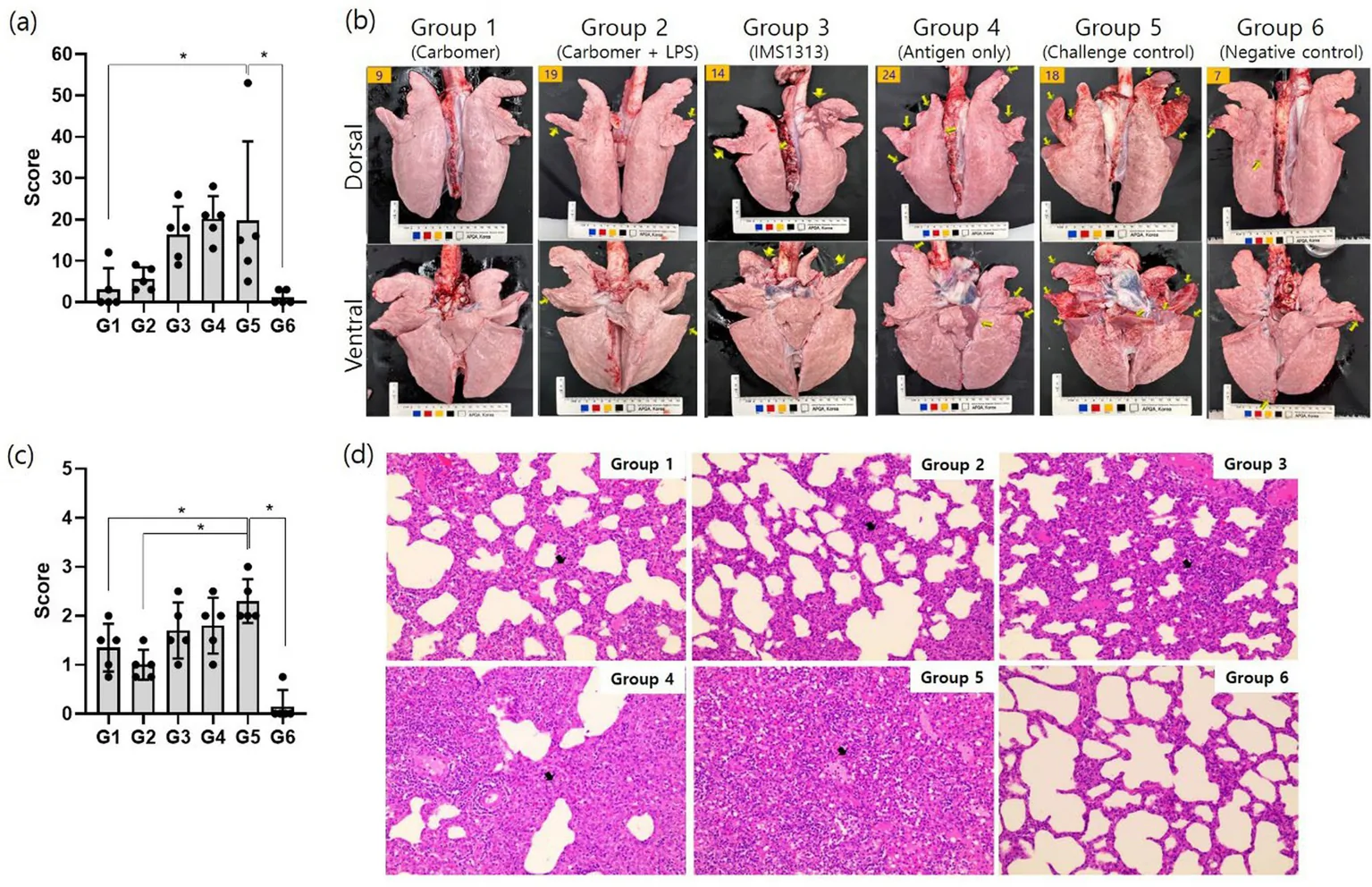
Macroscopic and histopathological analyses of lung tissues. **(a)** Lung lesion scores. Scores represent the percentage of affected lung volume (0–100%) using the Harbor method. G1 showed significantly lower scores than G5 (*p < 0.05), whereas G2 showed numerically lower scores compared with G5, but the difference was not statistically significant (1). **(b)** Representative microscopic images of each group. G5 showed typical PRRSV-associated lesions, including pulmonary consolidation and lung collapse failure, which were qualitatively alleviated in G1 and G2. **(c)** Comparison of histopathological lesion scores among groups. G1 and G2 exhibited superior reduction of pulmonary pathology against lung injury (*p < 0.05). **(d)** Representative histopathological images of lung sections (H&E staining) showing interstitial pneumonia (black arrow) and bronchial lesions.

## Discussion

This study evaluated the immunogenicity and disease attenuation of a homologous inactivated vaccine candidate against NADC34-like PRRSV-2, a variant that has recently caused significant economic losses in the domestic swine industry (27). Since its introduction into South Korea in 2022, the NADC34-like lineage has become a dominant strain, with higher detection rates and pathogenicity than other circulating lineages. However, research on vaccine strategies specifically tailored to this variant remains insufficient.

The present study investigates the protective efficacy of a high-dose inactivated vaccine against a homologous PRRSV genotype. Currently, the predominant PRRS vaccines in use are MLV vaccines. Although MLV vaccines are capable of mitigating viremia and improving daily weight gain, their inherent live-virus characteristics lead to critical field complications, including reversion to virulence and genetic recombination (28). Such risks highlight the urgent necessity for developing safer inactivated vaccines. Nevertheless, existing inactivated vaccines have historically demonstrated suboptimal protective efficacy, even against homologous strains (29). Here, we assessed the protective capacity of an inactivated vaccine formulated with a high antigen dose against a homologous challenge strain classified under Lineage 1 (Sublineage 1.5, NADC34-like). In standard efficacy trials for vaccine candidates, commercial vaccines are typically incorporated as reference controls. However, the only available commercial inactivated vaccine (Progressis) belongs to PRRSV-1 subtype 1a genotype. Because the genetic distance between PRRSV-1 and PRRSV-2 genotypes exceeds 40%, employing this commercial vaccine as a comparative control was deemed inappropriate for this study. Instead, by comparing a high-dose antigen-only cohort with groups utilizing diverse adjuvants, we successfully delineated the specific immunological contributions of the adjuvants and demonstrated their distinct efficacies relative to the challenge control group.

The experimental results showed improved clinical outcomes in groups vaccinated with a high-titer (10^8^ TCID_50_/dose) inactivated antigen, particularly when formulated with Carbopol/squalene-based adjuvants. In contrast to the nonvaccinated challenge control group (G5), which exhibited typical high fevers exceeding 41.0 °C, the Carbopol-adjuvanted groups (G1 and G2) showed significantly lower rectal temperatures and more rapid return to baseline during the post-challenge period. These findings suggest that, although the inactivated vaccine did not completely prevent viral infection, it may serve as an effective mitigation strategy by reducing post-infection clinical severity (14, 30).

A key finding of this study is that the protective effect of vaccination was more pronounced at the tissue level than in systemic indicators. Specifically, the Carbopol/squalene-based adjuvanted group (G1) exhibited a statistically significant reduction in pulmonary viral load and an improvement in histopathological interstitial pneumonia scores compared with the control group. These observations align with a previous study reporting that inactivated vaccines formulated with gel-based adjuvants can mitigate lung viral loads and pathological lesions in 3-week-old piglets, even in the presence of sustained viremia (24). The present findings suggest that by partially limiting viral infiltration and proliferation within lung tissue, the vaccine alleviated the primary respiratory damage characteristic of PRRS (27, 31). Notably, the absence of such protection in the antigen-only control group (G4) underscores the critical role of Carbopol/squalene adjuvants in eliciting localized immune responses to inactivated antigens.

A notable dissociation between outcome indicators emerged in this study: although the level of blood viral load did not differ significantly from those of the control group, lung lesion severity was markedly reduced. This finding suggests that blood-based viremia measurements, commonly used in PRRS vaccine efficacy evaluations, may not fully reflect disease mitigation at the tissue level (32). Therefore, future PRRS vaccine research should adopt multidimensional evaluation criteria that integrate both systemic and local pathological indicators.

Despite improvements in lung pathology, neutralizing antibodies were not detected in any of the vaccinated groups, and viral persistence in secondary lymphoid organs, including lymph nodes, remained unresolved. These findings highlight the limitations of current inactivated vaccine platforms in inducing sterilizing immunity (i.e., immunity that completely blocks systemic viral replication) (6, 7). The partial protection observed herein aligns with the current understanding of inactivated PRRSV-2 vaccines, particularly against emerging variants. Recent evaluations of NADC34-like strains have shown that both homologous inactivated and recombinant vaccine candidates primarily confer clinical and pathological mitigation rather than complete viral clearance (33). The present results support these reported limitations and reaffirm the technical challenges in overcoming the systemic immune-evasion mechanisms of contemporary PRRSV-2 lineages using inactivated platforms.

To evaluate the protective efficacy of the NADC34-like inactivated vaccine, pigs were challenged with a homologous variant. Interestingly, viremia levels at 7 dpc were observed to be lower in the challenge control group than in the vaccinated groups (Figure 5a and Supplementary Table 1) but no significant difference. This unexpected result raised the possibility of either a failure in vaccine-mediated viral reduction or an error during the challenge inoculation. However, a detailed analysis of individual efficacy parameters contradicts the likelihood of an inoculation failure. Following the challenge, the control group (G5) exhibited a significant febrile response (Figure 3a) and a reduction in average daily weight gain (Figure 3c) comparable to the vaccinated groups. Furthermore, while IgG antibody titers in the control group were lower at 4 dpc, this time point falls within the typical initial seroconversion window; by 7 dpc, the titers reached levels equivalent to those of the vaccinated groups (Figure 4a). The comparatively higher early antibody titers in the vaccinated cohorts are attributed to a rapid anamnestic IgG response stimulated by the prime-boost vaccination regimen. Tissue analyses also demonstrated that viral antigen loads in the tonsils and lymph nodes (Figure 6), along with lung lesion scores (Figure 7), were equivalent to those observed in the other groups. Collectively, these clinical and pathological findings sufficiently validate the pathogenicity of the challenge strain and confirm the success of the infection model. Nevertheless, large inter-animal variability in detection values or technical issues during the sampling collection may contribute to the results. Future studies should consider these issues and conduct further investigations.

Inactivated vaccines, by design, exhibit lower innate immune stimulation and generally induce weaker cell-mediated immunity than live vaccines (34). The partial protection observed in the carbomer-adjuvanted groups is attributable to the antigen depot effect of the adjuvant and to helper T-cell responses via dendritic cell activation (25). Furthermore, as reported by Shrestha et al. (2018), optimized vaccine formulations targeting mucosal or respiratory tract–specific immunity may help restrict viral replication at the site of entry, even when systemic clearance is not achieved. However, more potent immunostimulatory strategies remain necessary to achieve complete systemic viral clearance.

The Korean NADC34-like inactivated vaccine evaluated herein exhibited substantial efficacy in alleviating lung damage and clinical symptoms following infection with a highly pathogenic field strain. Although limitations remain in suppressing systemic viremia and achieving sterile immunity, the vaccine may serve as a tissue-level protective strategy against circulating domestic strains. Future research should focus on optimizing complex adjuvant systems or exploring prime–boost strategies combining live and inactivated vaccines to expand localized protection to systemic levels and enhance cell-mediated immunity (31, 34).

## Data Availability

The original contributions presented in the study are publicly available. This data can be found here: Genbank, accession numbers PZ169274, PZ169275.
