# Supplementary material for: A homologous NADC34-like PRRSV-2 inactivated vaccine partially modulates pulmonary viral load and pathological lesions under controlled challenge conditions
Source: Front Vet Sci. 2026 Oct 21;13:1932482. doi: 10.3389/fvets.2026.1932482 (PMC13732524; doi:10.3389/fvets.2026.1932482)
Supplement: Supplementary file 1 [file Data_Sheet_1.pdf]

A homologous NADC34-like PRRSV-2 inactivated vaccine partially modulates pulmonary viral load and pathological lesions under controlled challenge conditions

Su-Hwa You†, Hyun-Ji Lee†, Seung-Min Song, Eun-Gyeong Shin, Jae-Myung Kim, Yeun-Kyung Shin, Seok-Jin Kang*

Viral Diseases Research Division, Animal and Plant Quarantine Agency, 177, Hyeoksin 8-ro, Gimcheon, Gyeongsangbuk-do, 39660, Republic of Korea

Supplementary data

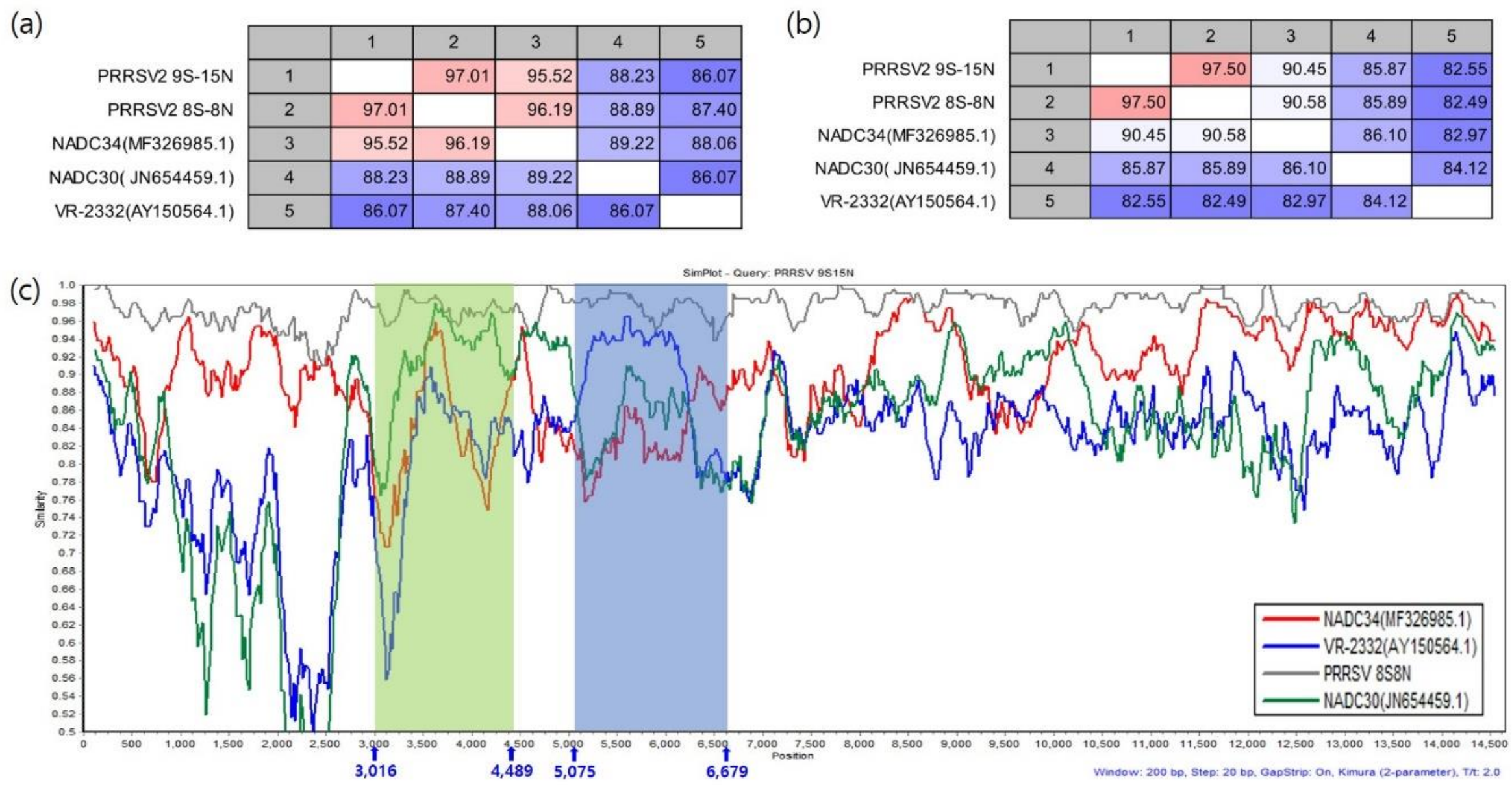

(a)

		1	2	3	4	5
PRRSV2 9S-15N	1		97.01	95.52	88.23	86.07
PRRSV2 8S-8N	2	97.01		96.19	88.89	87.40
NADC34(MF326985.1)	3	95.52	96.19		89.22	88.06
NADC30(JN654459.1)	4	88.23	88.89	89.22		86.07
VR-2332(AY150564.1)	5	86.07	87.40	88.06	86.07	

(b)

		1	2	3	4	5
PRRSV2 9S-15N	1		97.50	90.45	85.87	82.55
PRRSV2 8S-8N	2	97.50		90.58	85.89	82.49
NADC34(MF326985.1)	3	90.45	90.58		86.10	82.97
NADC30(JN654459.1)	4	85.87	85.89	86.10		84.12
VR-2332(AY150564.1)	5	82.55	82.49	82.97	84.12	

Supplementary Figure 1. Genetic characteristics of challenge stain, prrsv23-9s15n. The similarity of *ORF5* (a) and whole genome (b) of prrsv23-9s15n were compared with prrsv23-8s8n, NADC34, NADC30 and VR2332. (c) Recombination events detected in prrsv23-9s15n using RDP5 and SimPlot software. The recombinant positions of prrsv23-9s15n were similar with breakpoints in prrsv23-8s8n genome (Figure 2c).

Supplementary Table 1. Viremia kinetics post-challenge (mean ± SD).

	G1	G2	G3	G4	G5(PC)	G6(NC)
0dpc	0.0 ± 0.0	0.0 ± 0.0	0.0 ± 0.0	0.0 ± 0.0	0.0 ± 0.0	0.0 ± 0.0
7dpc	5034.3 ± 8968.3	10547.1 ± 20743.6	21228.6 ± 46700.2	21126.2 ± 42469.2	1854.4 ± 3658.9	0.0 ± 0.0
14dpc	422.3 ± 436.3	1583.9 ± 1355.5	3390.8 ± 4243.6	3333.4 ± 5266.8	4510.7 ± 5969.6	0.0 ± 0.0
21dpc	8.7 ± 19.4	55.0 ± 108.2	49.9 ± 58.8	44.5 ± 53.7	39.6 ± 54.5	0.0 ± 0.0
